# Unexpected detection of *Plasmodium vivax* and *Plasmodium falciparum* DNA in asymptomatic blood donors: fact or artifact?

**DOI:** 10.1186/1475-2875-13-336

**Published:** 2014-08-28

**Authors:** Alfredo Mendrone, Crispim Cerutti, José Eduardo Levi, Marcos Boulos, Maria Carmen Arroyo Sanchez, Rosely dos Santos Malafronte, Silvia Maria Di Santi, Vicente Odone

**Affiliations:** Fundação Pró-Sangue, Hemocentro de São Paulo, São Paulo, Brazil; Unidade de Medicina Tropical, Universidade Federal do Espírito Santo, Vitoria, Brazil; Faculdade de Medicina, Universidade de São Paulo, São Paulo, Brazil; Instituto de Medicina Tropical, Universidade de São Paulo, São Paulo, Brazil; Superintendência de Controle de Endemias, Secretaria de Estado da Saúde de São Paulo, São Paulo, Brazil

## Abstract

A study searching for *Plasmodium vivax* and *Plasmodium falciparum* DNA among blood donors from the non-endemic area in Brazil reported a rate of 7.41%. This number is at least three times higher than what has been observed in blood donors from the Amazon, an endemic area concentrating >99% of all malaria cases in Brazil. Moreover, the majority of the donors were supposedly infected by *P. falciparum*, a rare finding both in men and anophelines from the Atlantic forest. These findings shall be taken with caution since they disagree with several publications in the literature and possibly overestimate the actual risk of malaria transmission by blood transfusion in São Paulo city.

## Background

The recently published article from Maselli *et al.* [1] presents results and conclusions that shall be taken with caution, despite their sound and robust analytical method. In their article, the epidemiological knowledge of the “bromeliad-malaria” [2, 3] should have been taken into account. During the 1960s, malaria of simians was studied [4, 5] and, in Atlantic Forest, anophelines of the subgenus *Kerteszia* were incriminated as vectors of the two species of parasites considered equivalent to *Plasmodium vivax* and *Plasmodium malariae*. In contrast, *Plasmodium falciparum* was never detected in simians from the Atlantic Forest of the city of São Paulo [6]. Curiously, Maselli and co-workers state in the discussion that *“In the Atlantic Forest, Alouatta monkeys have been found to be infected by P. vivax and P. malariae [ref49], along with P. falciparum [ref48]”.* However, the cited work [7] does not involve any monkey sample and, in fact, previous studies on natural infection of anophelines from the Atlantic Forest of the states of São Paulo and Espírito Santo showed no *P. falciparum* infections in *Kersteszia* or *Nyssorhynchus* subgenus. Only *P. vivax* and *P. malariae* infections were detected [7–9]. Over the last two decades, several studies focused on various aspects of “bromeliad” malaria transmission [10, 11]. In some of those investigations, DNA supposed to be from *P. falciparum* was also amplified [10], but it did not usually resist to a second amplification by another method. Sometimes, the second amplification disclosed *P. malariae*, instead.

So far there are only two published studies investigating *Plasmodium* prevalence by molecular methods among Brazilian blood donors [12, 13], both performed in the endemic area (the Amazon) and cited in the paper of Maselli *et al.* [1]. The former described a 2.3% prevalence of *P. vivax* + *P. falciparum* infections, while the latter detected 1.34%. More than 99.9% of the reported Brazilian malaria cases come from this area so, obviously, more asymptomatic cases may occur there as well. How would this data conciliate with the 7.41% prevalence in São Paulo city and surroundings, as reported by Maselli *et al*.? What are the ecological conditions in São Paulo State that would sustain such a high rate of asymptomatic *P. vivax* and especially *P. falciparum* infections? The small number of transfusion-transmitted malaria cases (TTM) reported disagrees with the suggested high risk. There are only four cases published from São Paulo State [14–16], all connected to *P. malariae*, while no carrier of this species was reported by Maselli *et al*. Moreover, the PCR protocol referred by them (Gama *et al*. ref.#30) [17] is genus-specific whereas the primers employed in their real-time PCR assay are species-specific and most probably taken from the description of Perandin *et al.* [18], although there are a few nucleotide differences in the FAL-F and VIV-R primers.

It may be argued that the actual contribution of donors included in the “exposed” group to the total blood donors pool from Fundação Pró-Sangue/Hemocentro de São Paulo (FPS) is much less than the approximately 45% (500/1,108) enrolled in the study. This would justify a verified much lower rate of TTM than those numbers would predict. However, in an analysis of 17 spontaneous blood donors from Juquitiba (Atlantic Forest area, São Paulo state, one of the cities that directed donors to the “exposed” group in Maselli’s *et al.* work) that came to FPS, the ELISA using *P. vivax* recombinant antigen (ELISA-*Pv*) was reagent in 76% of samples and using *P. falciparum* total extract in 12%. SD Bioline test using recombinant antigens for both species (SD Bioline *Pf*/*Pv*) resulted positive in 71% for *P. vivax* and 0% for *P. falciparum*. This result points to a cross-reaction with *P. vivax* antibodies using *P. falciparum* total extract. One sample was positive for *P. malariae* by PCR [19]. Recently, samples of 39 donors from the same area were assayed employing a modified real-time PCR protocol [20] using genus-specific primers [17]. ELISA-*Pv* was reagent in 38% of samples and SD Bioline *Pf*/*Pv* was positive in 38% (*Pv* band) and 0% (*Pf* band). No sample was positive in real-time PCR (data not published).

The PhD thesis linked to this study is freely available [21] and it reports that ELISA was performed with the 84 samples found DNA+. Only eleven out of the 61 samples *Plasmodium* DNA + from the “exposed” group were seropositive, while none of the 23 from the non-exposed group was positive. This data is not included in the published manuscript. This huge discrepancy was never described in other cohorts. For example, 100% of blood donors in Nigeria were seropositive for *P. falciparum* [22], so it remains to be explained why in the São Paulo state cohort, donors that supposedly carry *P. falciparum* or *P. vivax* do not develop detectable antibodies.

The sample size was calculated based on the 1.3% prevalence of subclinical infection in blood donors in the Amazon region. However, this data is not available in the cited reference (ref.#3 Ministério da Saúde do Brasil: Sistema de Informação de Vigilância Epidemiológica – Malaria Sivep-Malária; 2010). The authors reported 146 autochthonous cases in 2013 in São Paulo State. In fact, data from Health Ministry registered 16 cases this year and the Health Secretary of São Paulo State informed 13 (provisional data). As the unique reference cited is from 2008 related to data from 1985 to 2006 (Marques *et al.* ref.#12), there is doubt about the source of these data. Additionally, the references #46 and #47 are improperly cited, since Scuracchio *et al.* show data from non-endemic area and Fugikaha *et al.* from endemic region.

## Conclusions

In conclusion, undoubtedly the issue of TTM in São Paulo State deserves further investigation, and certainly, there is an enhanced risk from donors with some epidemiological connection to the forested areas where *Plasmodium* (mainly *P. malariae* and *P. vivax*) persists, since currently there are no active measures to identify such asymptomatic carriers. Unfortunately, the work by Maselli *et al.*, instead of helping in clarifying the size of the risk and potential epidemiological associations in infected donors, brings confusion and unjustified alarm by presenting unrealistic data of donors carrying *Plasmodium* DNA.
